# Editorial: Strategies Played by Immune Cells and Mycobacteria in the Battle Between Antimicrobial Activity and Bacterial Survival

**DOI:** 10.3389/fimmu.2022.869692

**Published:** 2022-03-02

**Authors:** Veronica Schmitz, Eun-Kyeong Jo, Maria Teresa Ochoa, Rosane M. B. Teles

**Affiliations:** ^1^ Leprosy Laboratory, Oswaldo Cruz Institute, Oswaldo Cruz Foundation (FIOCRUZ), Rio de Janeiro, Brazil; ^2^ Department of Microbiology, and Infection Control Convergence Research Center, College of Medicine, Chungnam National University, Daejeon, South Korea; ^3^ Keck School of Medicine, University of Southern California, Los Angeles, CA, United States; ^4^ Division of Dermatology, Department of Medicine, University of California, Los Angeles, Los Angeles, CA, United States

**Keywords:** leprosy, mycobacterial diseases, cell death, cellular targets, immune evasion, autophagy, mycobacteria-host cell interactions

The interaction between host immunity and mycobacterial scape mechanisms determines the balance between antimicrobial activity and/or mycobacterial survival. This Research Topic, presented as original research and review articles, focused on mycobacteria-host cell interaction studies, including cellular and molecular targets of mycobacterial diseases, mycobacteria-mediated cell death mechanisms, host cell factors/pathways that act on mycobacteria to contain the infection, and immune evasion mechanisms utilized by mycobacteria (1–3).

The immune cells’ polarization and plasticity in response to mycobacteria are essential for an efficient innate and adaptative immunity. Ge et al. review the importance of macrophage diversity and polarization for the granuloma formation in mycobacterial diseases. Specifically, they focus on the importance of less studied types of macrophages such as multinucleated giant cells, epithelioid cells, and foamy macrophages. Nogueira et al. focused on the B cell diversity in response to mycobacterial infection analyzing the B cells subpopulations in the peripheral blood of patients across the leprosy spectrum. They found that patients with a high bacillary load have an alteration in the frequency and function of mature and memory B cells. A better understanding of the immune cell’s polarization and plasticity in response to mycobacterial infection is essential to preventing and treating mycobacterial diseases.

The activation of intracellular mechanisms by mycobacteria contributes to an efficient antimicrobial response, antigen presentation, and metabolic homeostasis. Kundu and Basu’s review article focused on the role of non-coding RNAs (miRNAs and lncRNAs) as immune regulators in host immune response to mycobacterial infection, acting in important pathways such as inflammation, autophagy, apoptosis, the polarization of macrophages, and mycobacterial survival. The presence of different non-code RNAs in the fluids of latent and active tuberculosis (TB) patients opens an important window for using non-coding RNAs as a therapeutic tool and as biomarkers of active and latent tuberculosis. In addition, Park et al. review the influence of host immune-mediated stresses, such as lysosomal activation, metabolic changes, oxidative stress, mitochondrial damage, and immune mediators, on the activities of the currently anti-TB drugs. Indeed, they also discussed how TB treatment facilitates the generation of *Mycobacterium tuberculosis* (Mtb) populations that are resistant to host immune response or disrupt host immunity. The long-term treatment may increase the risk of multidrug-resistant (MDR)- and extensively drug-resistant (XDR)-Mtb emergence.

Moreover, the ability of mycobacteria to inhibit phagolysosomal fusion also represents a key step in the determination of latent and active disease and can weaken the ability of the host immunity to fight against MDR- and XDR-Mtb. Saha et al. provide a new mechanism for mycobacteria evasion mediated by the macrophage at protein Coronin1 by the increase of intracellular cAMP levels upon mycobacterial infection. The increase of cAMP enhances cytoskeletal protein Cofilin1 to depolymerize F-actin around the mycobacteria-containing phagosome leading to the retarded phagosome maturation and acidification processes, providing mycobacterial survival. In their contribution, Zhou et al. showed that Mtb infection down-regulated GSK-3α/β activity and induced matrix metalloproteinase-1 (MMP-1) and -9 expressions in THP-1 derived-macrophages. MMP-9 protein expression was increased in the lungs of pulmonary tuberculosis and lymph nodes lymphatic tuberculosis patients, compared with that in patients of chronic inflammation. Their study has detected autophagy, pro-inflammatory and anti-inflammatory cytokines including IFNs and IFN stimulated genes (ISGs) upon Mtb infection with the treatment of SB216763, a GSK-3α/β inhibitor, in THP-1-macrophages. A better understanding of how mycobacteria interact with macrophage to block phagolysosome fusion and manipulate inflammation is important for the development of better therapeutic strategies.

An efficient antimicrobial response is critical for the clearance of mycobacteria inside of macrophages, metabolic response to hypoxia can regulate the expression and release of antimicrobial peptides. Zenk et al. showed the effects of one of the prolylhydroxylases inhibitors, Molidustat, which interferes with the interplay between Mtb and macrophages by interfering with the MAPK pathway *via* inhibition of p38 phosphorylation and stabilizes Mtb antigen mediated induction of hypoxia-inducible factor (HIF)-1α *via* TLR-signaling. These events lead to HIF-1α translocation into the nucleus and induction of the vitamin D antimicrobial pathway expression, resulting in the reduction of the proliferation of virulent Mtb in human macrophages. The study of how hypoxia can help to control intracellular pathogens including mycobacteria can be used as a new therapeutical strategy against mycobacterial diseases. Rasi et al. demonstrated that the enzymatic activity of Granzyme A (GzmA) is dispensable to mediate mycobacterial growth inhibition in primary monocytes infected by BCG. Global proteomic analysis of BCG-infected primary monocytes demonstrated that the ER stress response and ATP producing proteins were upregulated after GzmA treatment. These data raise the possibility for new targets for host-directed therapies to better control mycobacteria infections. According to the authors, future experiments are required to discern whether primary alveolar macrophages infected with Mtb recapitulate their findings.

The old antimycobacterial function of nitric oxide (NO) was revisited in this Research Topic. Zhang et al. highlight that sirtuin (SIRT) 7 contributes to limiting intracellular Mtb growth in RAW264.7 macrophages. SIRT7-mediated antimicrobial responses are partly due to NO production and apoptosis induction during Mtb infection. In addition, Ahn et al. presented the antimicrobial effects of recombinant interferon (IFN)-β for intracellular growth of *M. abscessus* infection. The pretreatment of recombinant type I IFN led to an increased NO production in the mouse lungs during *M. abscessus* infection, highlighting the function of the type I IFN-NO axis in the host defense against *M. abscessus*. Because cytotoxic NO action appears to be controversial in human monocytes/macrophages, future studies are warranted to clarify the function of SIRT7 and type I IFN in the context of NO regulation during the human antimycobacterial defense. In this Research Topic, Silwal et al. review the emerging position of autophagy in host defense against nontuberculous mycobacteria (NTM) infections. Although it is still in its infancy to understand the role of autophagy in various NTM diseases, compelling evidence suggests that autophagy-modulating strategies promote antimicrobial host defense and ameliorate pathological inflammation during several NTM infections. Together, these studies suggest that NO-inducing and autophagy-activating strategies may be curative therapeutic weapons to fight against TB and NTM infections.

This special Research Topic highlighted the innovative and review studies focusing on a complex interplay at the interface of mycobacteria and host factors, thereby counteracting mycobacterial pathogenesis and enhancing of protective host defense system. A deeper understanding of complex host-pathogen relationships will facilitate future investigations on this area for the development of potential candidates and the improved therapy against mycobacterial diseases.

## Author Contributions

The four authors reviewed and edited the manuscript. All authors contributed to the article and approved the submitted version.

## Conflict of Interest

The authors declare that the research was conducted in the absence of any commercial or financial relationships that could be construed as a potential conflict of interest.

## Publisher’s Note

All claims expressed in this article are solely those of the authors and do not necessarily represent those of their affiliated organizations, or those of the publisher, the editors and the reviewers. Any product that may be evaluated in this article, or claim that may be made by its manufacturer, is not guaranteed or endorsed by the publisher.
